# Metal–Insulator Transition of Ultrathin Sputtered Metals on Phenolic Resin Thin Films: Growth Morphology and Relations to Surface Free Energy and Reactivity

**DOI:** 10.3390/nano11030589

**Published:** 2021-02-26

**Authors:** Christian Schuster, Harald Rennhofer, Heinz Amenitsch, Helga C. Lichtenegger, Alois Jungbauer, Rupert Tscheliessing

**Affiliations:** 1 Austrian Centre of Industrial Biotechnology, Muthgasse 11, 1190 Vienna, Austria; rupert.tscheliessnig@boku.ac.at; 2 Department of Chemistry and Physics of Materials, Paris Lodron University Salzburg, Jakob-Haringer-Strasse 2a, 5020 Salzburg, Austria; 3 Institute of Physics and Materials Science, University of Natural Resources and Life Sciences, Peter-Jordan-Straße 82, 1190 Vienna, Austria; harald.rennhofer@boku.ac.at (H.R.); helga.lichtenegger@boku.ac.at (H.C.L.); 4 Institute of Inorganic Chemistry, University of Technology Graz, Stremayrgasse 9, 8010 Graz, Austria; heinz.amenitsch@elettra.eu; 5 Institute for Biotechnology, University of Natural Resources and Life Sciences, Muthgasse 18, 1190 Vienna, Austria

**Keywords:** thin films, polymer-metal interfaces, deposition, metal clusters, in situ resistivity, surface free energy

## Abstract

Nanostructured metal assemblies on thin and ultrathin polymeric films enable state of the art technologies and have further potential in diverse fields. Rational design of the structure–function relationship is of critical importance but aggravated by the scarcity of systematic studies. Here, we studied the influence of the interplay between metal and polymer surface free energy and reactivity on the evolution of electric conductivity and the resulting morphologies. In situ resistance measurements during sputter deposition of Ag, Au, Cu and Ni films on ultrathin reticulated polymer films collectively reveal metal–insulator transitions characteristic for Volmer–Weber growth. The different onsets of percolation correlate with interfacial energy and energy of adhesion weakly but as expected from ordinary wetting theory. A more pronounced trend of lower percolation thickness for more reactive metals falls in line with reported correlations. Ex situ grazing incidence small angle X-ray scattering experiments were performed at various thicknesses to gain an insight into cluster and film morphology evolution. A novel approach to interpret the scattering data is used where simulated pair distance distributions of arbitrary shapes and arrangements can be fitted to experiments. Detailed approximations of cluster structures could be inferred and are discussed in view of the established parameters describing film growth behavior.

## 1. Introduction

Many modern technologies rely on surfaces covered with nanometer-sized metal structures or thin metal films; most prominently optoelectronics, microelectronics, catalysis, energy conversion and storage, sensors and actuators and food packaging [1,2,3,4]. Also, emerging fields such as bio- and nanopore sensors [5,6], nanomedicine [7] and voltage-charging separations [8] rely on nanoscale metal structures. Many different physical surface effects such as surface plasmon resonance, surface-enhanced Raman scattering and metal-enhanced fluorescence [9] are enabling these applications. A considerable portion of the above technologies are based on assemblies where the substrate in contact with the metal structure is polymer-based. Defined properties of the respective polymer–metal assembly are often crucial for functionality where the metal structure and morphology is often the dominant determining factor. However, the nature of the emergent structures varies considerably with the fabrication technique or process parameters [10,11] and the underlying processes are often not fully understood. Key properties of the final system drastically depend on the structure of the metal coating where it is either a prerequisite that the coating is electrically conductive or that the deposited metal is present in separated clusters of a certain (often nanometer) scale.

In the past decade, ultrathin polymeric materials with large lateral dimensions have attracted attention because of their remarkable characteristics and potential applications in diverse fields of research and industry [12,13,14,15,16,17]. The potential applications overlap considerably with those of thin metal films in cases such as (bio-) sensors and actuators [13], microelectronics, energy conversion and storage [18], optoelectronics [19] and separations science [20,21]. Reticulated or covalently crosslinked polymer materials are particularly interesting owing to their high mechanical strength, dimensional stability as well as their chemical and thermal resistance [22,23]. Additional functionality or robustness can be imparted by metallization of either the surface or the whole volume of the film. Again, the resulting properties depend strongly on the type as well as the morphology of the involved materials [24,25]. In any event, the presence of the covalent polymer network is especially intriguing as it may prevent penetration of surface situated metal structures into the polymer or migration of fillers within the polymer even above its glass transition temperature [25,26]. On the other hand, metal implantation in compliant covalent polymer networks can be used to induce surface strain yielding tailorable buckling structures [27].

As a well-established process, physical vapor deposition and especially magnetron sputter deposition is used frequently [1] and at large scale [28] for the deposition of thin inorganic films. In a low-pressure atmosphere (typically argon), a confined plasma is generated by a high potential and the ionized nuclei are accelerated towards the material source (target) to knock out atoms which then travel to the substrate and form a solid film. Many processes, such as reemission, surface diffusion, implantation, bonding to the surface, coordination to existing clusters of sputtered material etc. are happening at the surface ultimately determining the shape and properties of the formed film. These kinetic processes and the implications for the emergent film characteristics when produced under clean ultrahigh vacuum conditions and on very defined inorganic substrates are reasonably well understood [29].

For the more specific case of polymer–metal interfaces the limited amount of available literature has been extended in recent years partly due to the advent of portable microelectronics and the associated strive for lightweight and flexible electronics [1]. Describing and predicting the behavior of metal–polymer interfaces is aggravated by several circumstances such as the intrinsically irregular shape and composition of the polymer surface. Adatom implantation may add another layer of complexity since it can result in altered mechanical properties and morphology of the surface or the metal-enriched zone [27]. In general, however, polymeric materials possess a low-surface free energy (SFE) as a result of low cohesion energy density. This can be rationalized by a comparatively low number of strong bonds per atom as opposed to more densely packed metals and metal oxides resulting in a high number of strong bonds per atom. Therefore, from this surface energy mismatch and thermodynamic considerations metals are expected to (and generally do) poorly wet polymeric surfaces. The analogy of liquids wetting solids implies that the interfacial energy will determine the shape of the droplets i.e., the metal clusters. Indeed, the difference in wetting behavior of gold sputter-coated on two different polymers has been inferred to correlate with the interfacial energy [30]. These results were substantiated by another study where a similar magnitude for the interfacial energy between gold and polymer was estimated based on geometries extracted from X-ray scattering data [3]. Silver thin films have also been shown to exhibit markedly different growth morphology with respect to substrate surface energy [31].

On the other hand, metals can form transient or permanent bonds with the polymer surface upon adsorption [32,33]. Reactive metals tend to bond to the surface more readily and will thus possess reduced mobility, resulting in less tendency to accumulate in clusters and thereby in better wetting [33]. For instance, more reactive metals evaporated onto several different polymers were shown to form interface morphologies strongly dependent on the nature of the metal [34]. Very frequently, metal coatings on polymers tend to evolve through different stages of non-layer-by-layer growth characterized by the presence of separated clusters that eventually undergo percolation to form a connected network after which the holes are gradually filled during the transition into a continuous film.

In this article, a systematic study of different metal films growing on ultrathin covalently crosslinked polymer films by direct current (DC) magnetron sputter deposition is presented. In order to gauge and facilitate the predictability of the emergent structures the film growth behavior is related to readily accessible material parameters. The metal thin-film formation of silver, gold, copper and nickel was studied by in situ resistance measurements where for all studied metals the resistance in the metal–insulator transition could be well fitted to the scaling law of an inverse Swiss-cheese percolation mechanism. With critical scaling exponents in the range 1.25–1.36, close to theoretical predictions [35,36], for all deposited metals a 3D island or Volmer–Weber growth mode can be inferred. Relations of the critical nominal film thickness of percolation (*d_c_*) to the respective energy of adhesion were found to correlate very weakly. A much clearer trend, which can be rationalized intuitively, is observed with the standard heat of metal oxide formation ΔH_f_^0^, a textbook parameter and proxy for metal reactivity, providing a valuable predictive guideline. Grazing incidence small-angle X-ray scattering (GISAXS) experiments complement our investigations and allow detailed approximations of the involved cluster geometries. By applying a new method to deduce experimental (real space) pair distance distributions (PDD), to which numerically calculated PDDs of model clusters and arrangements were fitted, we extracted geometric information. The models coincide well with the film growth behavior parameters obtained from established GISAXS analysis and might enable more a confident prediction of metal nanostructures based on surface free energy and metal reactivity.

## 2. Materials and Methods

### 2.1. Materials

Chloroform (CHCl_3_) and the polymers poly[(o-cresyl glycidyl ether)-co-formaldehyde] (PCGF, Mn = 870) and branched polyethylenimine (PEI, Mn = 10,000) were purchased from Sigma Aldrich (Vienna, Austria) and were used as received. The metal targets for sputter deposition were purchased from Gröpl (Tulln, Austria) with purities no less than 99.97%. The test liquids for contact angle measurements were HQ-H_2_O (0.055 μS cm^−1^), formamide (Roth (Karlsruhe, Germany), P040.1) and glycerol (Fisher Scientific (Vienna, Austria), BP229-1) and used as received.

### 2.2. Polymer Thin-Film Casting

Polymer thin films were fabricated on glass coverslips by spincoating with a WS650Mz-23NPP spincoater (Laurell, North Wales, PA, USA). Glass coverslips for casting were cleaned by immersion in concentrated sulfuric acid (96%) for at least two hours before they were thoroughly rinsed with HQ-H_2_O. After rinsing they were spun dry in the spincoater at 8000 rpm for 30 s. The polymer components PCGF and PEI were dissolved in chloroform to a concentration of 20 mg mL^−1^ and 10 mg mL^−1^, respectively. For the casting solution, 100 µL of the PCGF solution and 200 µL of PEI solution were mixed thoroughly and dropped on a freshly cleaned and dried cover slip. Covalent crosslinking of the resulting polymer film was achieved by placing the coverslip on a hot plate at 120 °C for 5 min.

### 2.3. Metal Thin-Film Deposition and Monitoring

A DC-magnetron sputter coater (SCD EM 005, Leica, Vienna, Austria) was used with Argon (99.999%) as the sputtering gas at a pressure of 0.9–1.2 × 10^−2^ mbar for metal thin film deposition. The plasma current was set to a value that resulted in a metal deposition rate between 0.06 and 0.08 nm s^−1^. The sputter current was roughly 15 mA for gold, 20 mA for copper and silver and 30 mA for nickel. The sputter targets were 48 mm in diameter resulting in overall power densities of 0.9, 1.2 and 1.8 W cm^−2^, respectively. The nominal film thickness and the deposition rate were monitored with a quartz crystal microbalance (EMQ SG100, Leica, Vienna, Austria) in situ with an accuracy of 0.1 nm and 0.01 nm s^−1^, respectively. In order to keep plasma-induced surface damage minimal the sample stage was positioned at the maximum distance of 10 cm from the target in all experiments. Furthermore, the sample and the microbalance were placed at the same eccentricity from the center (Figure S1). For ex situ experiments, after sputter deposition had been terminated the sample was left for another 30 s to let the measured thickness reach a constant value before ventilation of the vacuum chamber.

### 2.4. In Situ Resistivity Measurements

The electrical resistance of the growing metal films was measured with a LCR Meter ST2817A using a ST26011A Kelvin Clip Terminal (Sourcetronic, Bremen, Germany). The instrument was used in the R-X setting at a measurement frequency of 1 kHz and a measuring voltage of 1 V with open and short correction turned off. The resistance of the setup to contact the polymer surface was determined separately and subtracted from each measurement. The polymer surface was contacted via gold electrodes which were directly sputter-coated onto the surface through an appropriate mask to a thickness of 50 nm and a size of roughly 4 × 6 mm. The gold electrodes were connected to the copper wires with silver conductive adhesive with negligible electrical resistance. The contacts of the copper wires with the Kelvin clip terminal were checked for negligible resistance by a separate handheld LCR-meter. The samples were placed at the same perimeter as the microbalance during deposition to ensure an equal thickness of the deposited metal for at both locations (Figure S1).

### 2.5. Contact Angle Measurements

Contact angles (θ) of the respective test liquid (formamide, glycerol and water, see Materials) with the surface under investigation were measured in air with a DSA30 contact angle goniometer (Krüss, Hamburg, Germany) and evaluated with the provided software package Advance 3.0. Contact angles were recorded 10–60 s after drop deposition (depending on the liquid viscosity) to allow the droplet to reach force equilibrium and a static value of θ. The average value of both observed angles in each image was recorded. Contact angles were determined as the average value of at least five individual droplets (0.5–3 µL) on each of two individually prepared surface samples.

### 2.6. Grazing Incidence Small-Angle X-ray Scattering (GISAXS) Measurements

Grazing incidence small-angle X-ray scattering experiments were conducted with the respective metal coated polymer film as produced by spin coating and subsequent sputter coating without further treatment. The photon energy was 8 keV (λ = 0.154 nm) in all experiments. Thin films of gold, nickel and silver were investigated at the Austrian SAXS Beamline at the Elettra Synchrotron facility. Scattering patterns were recorded at room temperature with a Pilatus 1M detector (Dectris, Baden-Daettwil, Switzerland) with an exposure time of 10 s. For gold samples of 1, 3, 5 and 7.5 nm nominal thickness, the sample to detector distance (D) was 1403 mm (with a resulting q-range from 0.055 to 3.02 nm^−1^) and the incident angle was α_i_ = 0.24°. For gold samples with a nominal thickness of 9 and 12 nm, D was 1343 mm (with a resulting q-range from 0.08 to 3.50 nm^−1^). For silver and nickel, D was set to 1395 mm (q-range from 0.06–3.28 nm^−1^) and the incident angle was α_i_ = 0.68° and α_i_ = 0.625°, respectively. The copper films exhibited very high resistivity as they were checked at the synchrotron facility presumably due to oxide formation. Therefore, a separate set of copper samples were investigated shortly after deposition with a laboratory X-ray light source (S-Max 3000 with MM002+ Cu Kα source and a Triton200 multiwire detector, Rigaku, Tokyo, Japan). The incident angle was α_i_ = 0.20° and D was 1500 mm (q-range 0.1–2.7 nm^−1^). Horizontal line cuts of the scattering data (along the *y*-axis) were made at the Yoneda intensity for all samples with a pixel width of at least 5 to reduce noise for the low intensity, high q-range data.

## 3. Results and Discussion

### 3.1. Polymer Surface and Thin-Film Growth

For the investigation of metal film growth on a covalently crosslinked polymer surface an epoxy resin was used as an ultrathin film. Such ‘nanomembranes’ have been introduced as very robust polymer films in a free-standing fashion [22]. They were shown to retain their electrically insulating behavior for thicknesses down to 30 nm and can be fabricated to a sufficiently large scale with a facile spin-coating process. For this work, the polymer thin films were directly cast onto glass coverslips and covalent crosslinking was initiated at elevated temperature [22].

**Figure 1:**
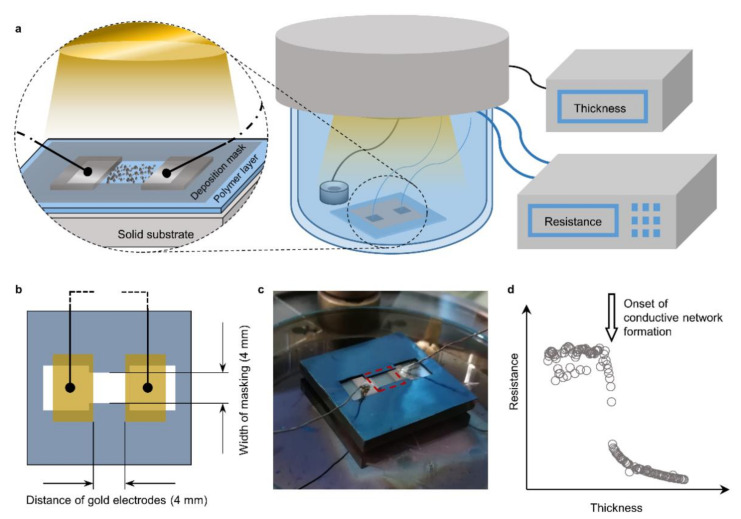
In situ resistance measurement setup. (**a**) Schematic representation of the general setup (right side) with a detailed representation of the sampling area; (**b**) geometry of the growing film between the gold electrodes confined by the masking; (**c**) optical image of a contacted sample sitting on the sample stage. The 4 × 4 mm area of measured thin film is indicated by a red dashed rectangle; (**d**) typical results obtained by relating the resistance to the thickness both measured in situ.

Metal thin films were deposited with a DC-magnetron sputter coater with Argon (99.999%) as the sputtering gas at a pressure of 0.9–1.2 × 10^−2^ mbar. The nominal thickness (d) of the growing film was monitored with a quartz crystal microbalance. For the investigation of the resistance (R) of the thin films, in situ resistance measurements were conducted during sputter deposition (Figure 1a). 

**Figure 2:**
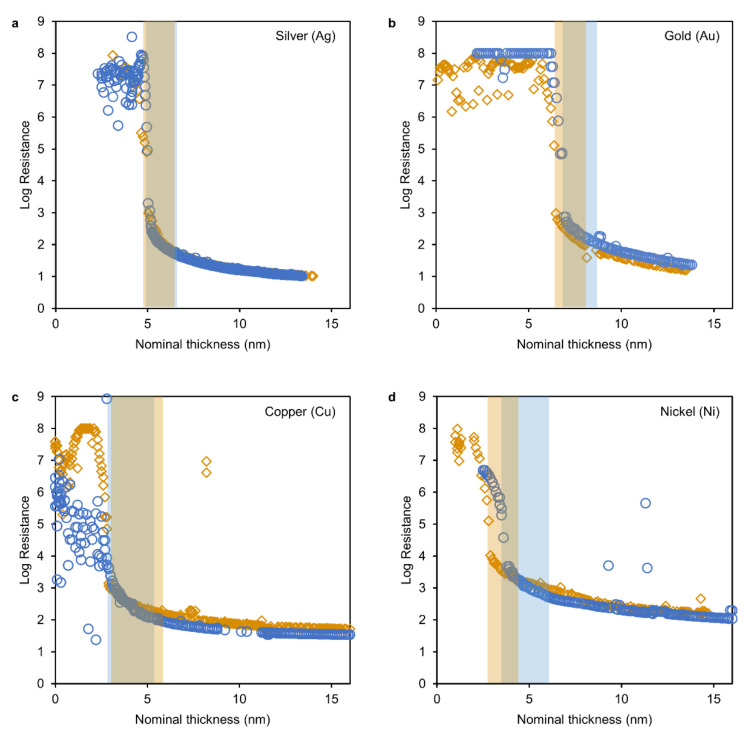
Results of in situ resistance measurements plotted as resistance versus nominal film thickness for Silver (**a**), Gold (**b**), Copper (**c**) and Nickel (**d**). Regions where equation 1 was fit to the data are shaded in the respective color.

To this end, the pristine polymer films were first sputter-coated with two 50 nm thick rectangular gold patches separated by a 4 mm gap (Figure 1b). These patches of gold served as electrodes and were contacted to an LCR-meter with copper wires. The ultrathin metal films were grown on a 4 mm wide strip between the two gold electrodes while the rest of the polymer surface was masked. This resulted in a 4 × 4 mm [2] thin film growing between the electrodes that is being measured for its resistance (Figure 1b,c). The growth rate was maintained within 0.06–0.08 nm s^−1^ for all metals by adjusting the potential and the resulting plasma current. The resistance was thereby obtained directly as sheet resistance (R_□_, Ohm per square) and correlated to the corresponding nominal film thickness for each experiment as in Figure 1d. For each metal, two experiments were conducted (Figure 2) and the resulting metal insulator transition in the R_□_ vs. d relation was fit to the function

(1) $$R_{□}=A*{\left(d-d_{c}\right)}^{-t}$$

where *d_c_* is the critical thickness, t is the universal critical exponent and A is a constant of proportionality. The resulting parameters are summarized in Table 1 as average values and their corresponding sample range.

All transition regions (as marked by the shaded areas in Figure 2) could be fitted to the above relation. The lower and upper boundary were the inflection point of the ‘sigmoidal’ shape of the data (around 10^5^–10^6^ Ohm) and the onset of a decline in fit quality (increasing average error) upon a widening of the fit region (starting from 2 nm width), respectively. The critical exponents were found to range from 1.24 to 1.37, which is in close proximity to theoretical predictions (1.34) [35] and experimental values of model percolation systems (1.29 and 1.34) [37]. These systems are characterized by the growth of conducting entities (clusters) on a non-conducting plane or in a non-conducting space which undergo percolation to form a conducting network. This is equivalent to metal clusters growing on a non-conducting substrate.

Thus, the acquired data indicate that in all experiments the thin film formation progressed from nucleation of clusters to a stage of coalescence and through the percolation into a conducting network to a final uniform film (Volmer–Weber growth). Note the fluctuating values of high resistance before the onset of conductivity in the low thickness regions. This conductivity can be attributed to the plasma which is present in the deposition chamber as these low resistances were only observable during the deposition and no conduction was observable when the deposition was interrupted. Also note the comparatively low resistance in the region before percolation during one measurement of copper (Figure 2c, blue circles). This resulted from using a specific measurement range of the LCR-device before changing to automatic shortly before the transition region.

### 3.2. Wetting, Percolation and Surface Free Energy

For thin metals applied to inorganic substrates in a low-pressure atmosphere a thermodynamic argument can be made to estimate the degree to which the metal wets the surface [28]. The picture is equivalent to the theory describing the wetting of surfaces by liquids where the surface free energy (SFE) and the interfacial energy between the involved phases determine the extent of spreading of the liquid on the surface. These material parameters have been tabulated in great detail for most metals and their behavior on defined inorganic surfaces has been studied extensively. The surface free energy is arguably a very suitable choice as a material characteristic to correlate with the observed onsets of conduction and thin film growth in general. Indeed, a difference in surface free energy behavior of the coated surface has been linked to a change in growth behavior [30,31,38].

For the polymer surface it stands to reason that the SFE is regarded and estimated separately, due to the inherent inhomogeneity and in view of the specificity of the fabrication and testing conditions. To this end, the static contact angle method has been applied here in conjunction with the by Good–van Oss–Chaudhury theory. This theory distinguishes between three components that surmount to the overall surface free energy (γ) which stem from the contributions of dispersive Lifshits–van der Waals (LW) and positive (+) as well as negative (−) polar interactions. Using the test liquids water, glycerol and formamide and solving the resulting three equations simultaneously [39] a value of $\gamma_{P}$ = 25.4 mJ m^−2^ ($\gamma_{P}^{LW}$ = 21.4 mJ m^−2^, $\gamma_{P}^{+}$ = 1.3 mJ m^−2^, $\gamma_{P}^{-}$ = 3.2 mJ m^−2^) for the SFE of the polymer (subscript P) was estimated (Supplementary Materials Section 2 and Table S1). Furthermore, it might be similarly worthwhile to question whether the surface free energy of the bulk metal is an adequate measure to be related to the experimental polymer SFE values. On the one hand, SFE values are very prone to fluctuations (e.g., different laboratory, operator, etc.) and are, therefore, notoriously difficult to reproduce and compare. Therefore, values obtained in a self-consistent manner under similar conditions for all involved materials will be strongly favorable. On the other hand, it is intuitive that, compared to the bulk metal, the sputtered metal will be well relaxed due to surface diffusion and will therefore have a lower surface free energy. Indeed, by applying the same methodology as for the polymer surface, SFE values for the sputtered metals ($\gamma_{M}$) have been estimated as shown in Table 2 and are found to be substantially lower than values reported for bulk metal. Note that the contact angle values were obtained immediately after sputter deposition to keep surface contaminations minimal. We found significant drift of the contact angles when obtained after extended exposure to ambient atmosphere, presumably due to airborne organic contaminants (compare Tables S1 and S3). SFE values obtained for gold are of the same order of magnitude as those found by Ruffino et al. [30] whereas for the other elements we found no references reporting values on sputter-deposited metal surfaces. Using the relation:

(2) $$\gamma_{M/P}={\left(\sqrt{\gamma_{M}^{LW}}-\sqrt{\gamma_{P}^{LW}}\right)}^{2}+2\left(\sqrt{\gamma_{M}^{+}*\gamma_{M}^{-}}+\sqrt{\gamma_{P}^{+}*\gamma_{P}^{-}}-\sqrt{\gamma_{M}^{+}*\gamma_{P}^{-}}-\sqrt{\gamma_{P}^{+}*\gamma_{M}^{-}}\right)$$

the interfacial free energy between the polymer surface and the sputtered metal ($\gamma_{M/P}$) was estimated from their respective SFE. Additionally, according to the following relation the corresponding adhesion energy ($E_{adh}$) can be estimated [29]:

(3) $$E_{adh}=\gamma_{M}+\gamma_{P}-\gamma_{M/P}$$

The values estimated for the different metal–polymer pairs are listed in Table 2.

**Figure 3:**
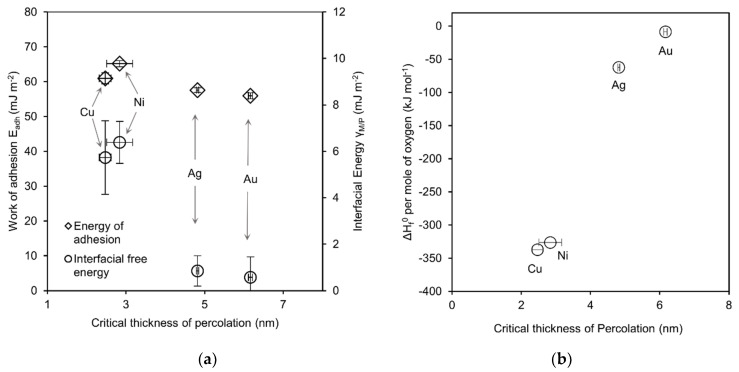
Plots of (**a**) energy of adhesion (*E_adh_*) and interfacial energy (*γ_M/P_*) and (**b**) standard heat of metal-oxide formation (ΔH_f_^0^) per mole of oxygen versus critical thickness of percolation for the investigated elements as indicated. Vertical error bars represent standard deviation, horizontal error bars represent sample range of *d_c_*.

In Figure 3a both parameters ($\gamma_{M/P}$ and $E_{adh}$), when plotted versus the critical percolation thickness, reveal a trend of decreasing energy with increasing thickness of percolation. Better wetting is expected for interfaces with higher energy of adhesion, since it reflects the work needed to separate the interface whereby higher values represent better adhesion. The estimated values for $E_{adh}$ show a slight trend in accordance with this expectation. However, this correlation is very weak compared to the absolute values and might be regarded as lying within the estimation uncertainty. According to Equation 2 the interfacial free energy $\gamma_{M/P}$ influences the work of adhesion so that this parameter is expected to correlate with the wetting behavior as well. Indeed, in terms of *d_c_* the wetting behavior displays a pronounced trend with the interfacial free energy. However, the expected correlation would be that interfaces with higher free energy are less favorable and better wetting would be predicted for low energy interfaces which is the opposite trend as observed. Nevertheless, the values of the interfacial free energies are quite small compared to the surface free energy of the involved materials so that its influence on $E_{adh}$ remains limited. Overall, the weak correlation might be attributed to other effects that can be neglected on the macroscopic scale but are significant at very small length scales. Thus, classical surface free energy contributions can be overshadowed resulting in the limited applicability of this concept to describe the percolation behavior.

### 3.3. Reactivity

Metal adatoms that impinge on the polymer surface might form bonds and thereby fix their location prohibiting surface diffusion. As a result cluster formation and coalescence will be diminished depending on the strength of the bond. It has been shown that metals can induce decomposition of the polymer [34] and strong bonds are formed even in non-reactive systems [40]. In consequence, it is intuitive that more reactive metals tend to form more defined interfaces and reach percolation earlier unless the growth proceeds in a layer-by-layer fashion to begin with. As a measure of reactivity, the standard heat of formation (ΔH_f_^0^) of the respective most stable metal-oxide as found in textbooks [41] was plotted versus *d_c_* in Figure 3b. A strong correlation can be observed where metals exhibit lower critical percolation thicknesses with increasing reactivity. Note that this observation is intuitive since interfacial bonding should decrease the mobility of metal adatoms and clusters which, in turn, reduces the ability to coalesce into larger particles and thereby lets the film undergo percolation at an earlier stage. The reactivity parameter used here was also reported to show correlations on selected inorganic substrates but broke down severely for others [29]. Thus, an extensive study covering more metals ideally on different polymer surfaces could help to verify the reliability and shed light onto the dependencies of this relationship providing valuable information and guidance for the design of polymer–metal interfaces.

### 3.4. Cluster and Film Morphology

**Figure 4:**
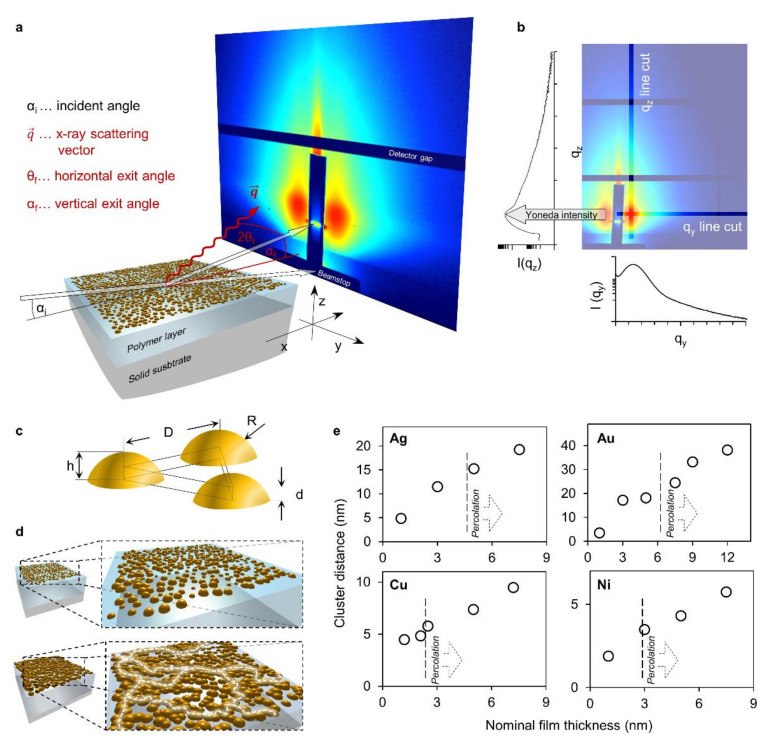
Grazing incidence small-angle X-ray scattering (GISAXS) experimental setup with line cuts, approximate cluster geometries and characteristic cluster distances; (**a**) schematic representation of the experimental setup used to obtain 2D X-ray scattering patterns at grazing incidence; (**b**) example of a 2D scattering pattern where the positions of cuts along the y- and the *z*-axis are indicated while the resulting scattering curves are plotted below and left of the pattern, respectively. The position of the Yoneda intensity, the z position where cuts along the *y*-axis are made, is indicated; (**c**) schematic representation of an idealized equilateral triangular unit cell of spherical cap shaped clusters; (**d**) conceptual progression of isolated clusters of metals percolating into a conductive network. Sufficient disorder results in irregularly shaped clusters and the formation of conductive paths as indicated by white dashed lines; (**e**) the distance estimated by Kratky analysis of the prominent scattering features caused by the interference function of the cluster arrangement plotted versus the nominal thickness. The respective metal is indicated in the upper left corner. The respective percolation thresholds and onsets of conduction are indicated by dashed lines.

The wetting behavior and onset of conduction will be tightly linked to the geometry of the metal structures present during growth. Furthermore, the number, size and geometry of the metal clusters is of vital importance for the emergent catalytic [42,43] or optical properties [44]. In order to gain an insight into the involved geometries, GISAXS experiments of the metal thin films were performed at various different thicknesses. A conventional experimental setup (Figure 4a) was used to obtain 2D scattering patterns of the metal coated polymer films. The diffuse out of plane scattering intensity as a function of the scattering vectors along the *y*-axis at the Yoneda intensity [45] (Figure 4b) contains information of the lateral sample morphology (q_y_-cuts). As established above by the thickness-resistivity behavior, the investigated metal coatings are progressing from isolated metal islands towards continuous films.

Certain observable characteristic features of the scattering curves (maxima or shoulders, see Figure S4) can be ascribed to the interference function, which reflects the cluster positions as they are arranged on the surface with a certain degree of order. To extract geometric information about the isolated clusters on the surface it is very common to approximate their shape as spherical caps and their assembly with a hexagonal lattice. This can be simplified to an equilateral triangular unit cell (Figure 4c). Given sufficient size and disorder of the metal clusters it is conceivable that they will eventually percolate to form a conductive network well before a close-packed arrangement is established (Figure 4d). However, metal clusters that are not entirely (hemi) spherical in shape but display an elongated or frayed shape causing an earlier onset of percolation might be expected. In spite of a certain disorder, for such an arrangement of clusters a characteristic average distance D to the nearest neighbors can be readily analyzed from Kratky plots of the q_y_-data [46] (Figure S2). The q_y_-positions of Gaussian functions, fit to the characteristic peaks that stem from the interference function, give an estimate of the average cluster distance D (Figure 4c). Furthermore, this estimated cluster distance can be utilized to discriminate between different growth regimes based on its rate of change with nominal thickness [19,38,47,48]. The evolution of this parameter for each metal (Figure 4e) can be interpreted to be subject to such a change. As indicated, the transitions to another rate of change coincides, most notably for copper and gold, with the onset of percolation at the critical thickness *d_c_*. In fact, an additional transition should be visible for the crossover from nucleation to coalescence [47] at low nominal thicknesses which is, however, not resolved by the present investigations.

**Figure 5:**
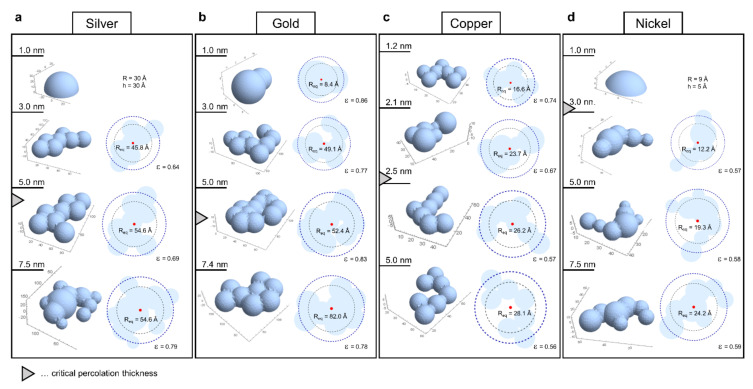
Approximated cluster geometries for GISAXS thin-film investigations of Silver (**a**), Gold (**b**), Copper (**c**) and Nickel (**d**). In each panel the cluster models are presented from top to bottom for successively thicker films as indicated on the left-hand side. The 3D representations of the estimated cluster geometries are shown left and the top down projections with the radius of gyration (R_g_, thin black dashed circles, value not shown) and the radius of a sphere with equivalent R_g_ (R_eq_, thick blue dashed circles) are shown on the right side. ε is the ratio between cluster footprint area within the blue circle and the whole area within that circle and indicates the compactness.

In order to further investigate the morphology we apply a method that is not restricted to a single form factor such as a spherical cap. Instead, we use the horizontal scattering data (q_y_) to compute pair distance distributions (PDD), the real-space equivalents of the scattering curves [49], to generate model cluster structures and arrangements. To compute the Fourier transform for systems of dimension D we relate the scattering intensity $I\left(Q\right)$ and the PDD by $\;I\left(Q\right)\propto F_{D}\left(PDD\left(r\right)\right)\left[Q\right]=\int_{0}^{\infty }PDD\left(r\right)drJ_{D/2-1}\left(Qr\right)/{\left(Qr\right)}^{D/2-1}$. It is the pair distance distribution of potential scattering sites in the sample that determines the scattering signal. Consequently, we can analyze the in plane scattering intensity (q_y_) by letting D = 2 whereby the Fourier transform is given by $\;F_{2}\left(PDD\left(r_{y}\right)\right)\left[Q\right]=\int_{0}^{\infty }PDD\left(r_{y}\right)dr\;J_{0}\left(qr_{y}\right)$ which means that it is given by the Bessel zero transform of the pair distance distribution. By discretizing $J_{0}\left(qr_{y}\right)$ and $PDD\left(r_{y}\right)$ on a finite interval and using a linear programming algorithm, through $PDD\left(r_{y}\right)dr\;J_{0}\left(qr_{y}\right)$ the Fourier transform $F_{2}\left(PDD\left(r_{y}\right)\right)\left[Q\right]$ that best satisfies the respective scattering data $I\left(Q\right)$ can be obtained by numerical minimization of their $L^{2}$ norm according to $min∥I\left(Q\right)-F_{2}\left(PDD\left(r\right)\right)\left[Q\right]{∥}_{2}$ (Supplementary Materials Section 4, Figures S3 and S4). Thereby ‘experimental PDDs’ are obtained (Figure S3). Finally, PDDs of model clusters were compared to these experimental PDDs to obtain representative cluster shapes as shown in Figure 5.

For the analysis of each experimental PDD, first, a large set of randomly shaped cluster models was generated using a set of appropriately chosen parameters. For each model in the random set its corresponding PDD was estimated using a Monte Carlo approach and the root mean square distance to the experimental PDD was calculated. The most representative models were then obtained as those giving minimal distance to the experimental PDDs resulting in an estimate of the average cluster shape and size in the samples. In Figure 5, representative cluster models are reported in terms of the respective radius of gyration (R_g_, black dashed circles). Additionally, the circumferences of (half)spheres with an equivalent R_g_ are superimposed as thick blue dashed circles with radius R_eq_. The elongation and branching (or conversely the compactness) of the clusters is indicated by the parameter ε. It is the ratio between cluster footprint within their spheres of equivalent R_g_ and the footprint of that sphere (i.e., ratio between the light blue area within the blue dashed circle and the area encompassed by that circle).

For all four metals a consistent trend of increasing cluster size with increasing film thickness was found. However, considerable differences in cluster size with respect to the different metals can be observed with gold exhibiting the largest and nickel the smallest average cluster size. Cluster shapes evolve from mostly spherical at the lowest thickness to more irregular, elongated and branched. This is indicated by initially high values of ε declining towards percolation and again higher values for thicker films. This aligns well with the notion of incomplete coalescence after a certain thickness and the filling of gaps after percolation. Interestingly, gold clusters at 1.0 nm thickness (Figure 5b) were estimated to exhibit the smallest radius (R_eq_ is the radius of a sphere with equivalent radius of gyration, see Supplementary Materials Section 7). This can be rationalized by adhesion energy and the low tendency for bonding so that gold clusters form the smallest interface area and most spherical clusters. Spheres accommodate more material than shallow clusters with identical radius and are accordingly smaller. All other geometries at 1 nm thickness must be shallower to fit the experimental PDDs. It is also worth noting that the geometries of silver and gold clusters, which exhibit the highest onsets of percolation, display markedly more compact and circular footprints with ε values around 0.7–0.8 (Figure 5a,b) than those of copper and nickel with ε values near 0.6 (Figure 5c,d). This is in accordance with the notion that more spread out and branched structures undergo percolation earlier as well as the concept of adhesion energy determining the tendency to form an interface with the surface. It also falls in line with higher metal reactivity diminishing cluster mobility by bonding to the surface since mobility is needed to relax into a spherical geometry. More resulting morphologies are discussed in detail in Supplementary Materials Section 8.

Cluster arrangements were also fit to the experimental PDDs while approximating clusters as spheres for simplicity (Supplementary Materials Section 8). The characteristic distance estimated from the interference function by Kratky analysis served as a reference to validate both the determined experimental PDDs and the applied models. Arrangements were found to exhibit the highest order at low thicknesses as order peaks gradually vanished with increasing thickness. Conveniently, the generic triangular unit cell of spherical caps (Figure 4c) allows the validation of the inferred geometry by the formulation of a simple conservative mass balance. The entire volume of material deposited on the triangle to height d (i.e., the nominal thickness) must be contained within clusters of the respective geometry (i.e., spheres for cluster arrangement fits). Since the cluster arrangement fits take into account the entire scattering curve they will be more or less (information of q_z_ is entirely disregarded) quantitative. Indeed, we found that arrangement fits with reasonably preserved conservative mass balance were obtainable for low nominal thicknesses. This indicates that cluster geometries are indeed most spherical at low thicknesses and can, therefore, be reasonably approximated by spheres. For higher thicknesses, however, considerable discrepancies were observed. For these, the mass balance might be satisfied to some extent by small clusters interspersed randomly between the large ones. The remaining discrepancy may be due to (i) the loss of cluster coupling caused by increasing disorder and an associated more diffuse scattering as well as (ii) the increasing cluster distance such that information is lost due to resolution and q-range limits. Nevertheless, the fitted arrangements yielded average nearest neighbor distances that agreed well with the values obtained from the widely used Kratky plots.

## 4. Conclusions

The growth of thin films of copper, gold, nickel and silver on covalently crosslinked polymeric substrates could be shown to weakly correlate with metal surface free energy but to strongly correlate with metal reactivity. This means that the ordinary Good–van Oss–Chaudhury wetting theory is not suitable to predict the film evolution of these systems. On the other hand, reactivity parameters are readily available as tabulated data and could serve in the straightforward prediction and tuning of nanostructured metal–polymer interface properties and their application performance. The combination of the observable correlations with the structural information deduced from GISAXS experiments should further aid in the rational design as well as the optimization of functional materials employing nanostructured polymer–metal interfaces.

## Figures and Tables

**Table 1:** Critical thickness of percolation and critical exponents found for in situ resistance measurements. Reported values are the average ± the range values of the two respective experiments.

Metal	*d_c_* (nm)			t		
Gold	6.2	±	0.1	1.32	±	0.01
Silver	4.8	±	0.1	1.27	±	0.03
Copper	2.5	±	0.2	1.31	±	0.06
Nickel	2.9	±	0.3	1.30	±	0.01

**Table 2:** Estimated surface free energy components, interfacial energy (*γ_M/P_*) and adhesion energy (*E_adh_*) for the polymer surface and the respective metal reported as average and standard deviation.

Surface	γ			γ^LW^			γ^+^			γ^-^			γ_M/P_			E_adh_		
	(mJ m^−2^)																	
Polymer	25.4	±	0.7	21.4	±	0.4	1.3	±	0.3	3.2	±	0.2						
Ag	33.0	±	1.3	23.1	±	0.6	4.6	±	0.6	5.5	±	0.5	0.8	±	0.7	57.6	±	1.1
Au	31.1	±	2.2	16.8	±	1.3	3.4	±	1.2	14.8	±	1.2	0.6	±	0.9	56.0	±	1.0
Cu	41.3	±	2.0	15.7	±	0.9	6.0	±	0.8	27.4	±	0.8	5.7	±	1.6	61.0	±	0.3
Ni	46.2	±	0.4	20.8	±	0.2	7.2	±	0.2	22.2	±	0.1	6.4	±	0.9	65.2	±	0.1

## Data Availability

The data presented in this study are available on request from the corresponding authors.

## Supplementary Materials

The following are available online at https://www.mdpi.com/2079-4991/11/3/589/s1: Section 2: Contact angles and surface free energy (SFE) derivation, Section 4: Experimental pair distance distributions (PDD) computation, Section 7: PDD calculation and fitting, Section 8: PDD fits and morphology, Figure S1: Sputtering chamber setup, Figure S2: Kratky plots and analysis, Figure S3: Experimental PDDs, Figure S4: Raw q_y_ scattering data and experimental PDD fits, Figure S5: Monte Carlo approach for numerical PDD calculation, Figure S6: PDD cluster fits for silver, Figure S7: PDD cluster fits for gold, Figure S8: PDD cluster fits for copper, Figure S9: PDD cluster fits for nickel, Table S1: Contact angle data, Table S2: Surface energy of test liquids, Table S3: Contact angle data after 24 h.
