# Supplementary material for: Metal–Insulator Transition of Ultrathin Sputtered Metals on Phenolic Resin Thin Films: Growth Morphology and Relations to Surface Free Energy and Reactivity
Source: Nanomaterials (Basel). 2021 Feb 26;11(3):589. doi: 10.3390/nano11030589 (PMC7996922; doi:10.3390/nano11030589)
Supplement: Supplementary file 1 [file nanomaterials-11-00589-s001.pdf]

Supplementary Material

Metal–Insulator Transition of Ultrathin Sputtered Metals on Phenolic Resin Thin Films: Growth Morphology and Relations to Surface Free Energy and Reactivity

Christian Schuster [1,2,*], Harald Rennhofer [3], Heinz Amenitsch [4], Helga C. Lichtenegger [3], Alois Jungbauer [1,5,*] and Rupert Tscheliessing [1]

[1] Austrian Centre of Industrial Biotechnology, Muthgasse 11, 1190 Vienna, Austria; rupert.tscheliessnig@boku.ac.at
[2] Department of Chemistry and Physics of Materials, Paris Lodron University Salzburg, Jakob-Haringer-Strasse 2a, 5020 Salzburg, Austria
[3] Institute of Physics and Materials Science, University of Natural Resources and Life Sciences, Peter-Jordan-Straße 82, 1190 Vienna, Austria; harald.rennhofer@boku.ac.at (H.R.); helga.lichtenegger@boku.ac.at (H.C.L.)
[4] Institute of Inorganic Chemistry, University of Technology Graz, Stremayrgasse 9, 8010 Graz, Austria; heinz.amenitsch@elettra.eu
[5] Institute for Biotechnology, University of Natural Resources and Life Sciences, Muthgasse 18, 1190 Vienna, Austria
* Correspondence: Christian.Schuster@sbg.ac.at (C.S.); alois.jungbauer@boku.ac.at (A.J.); Tel.: +43-1-47654-79083 (C.S.)

1. Sputtering Chamber Setup

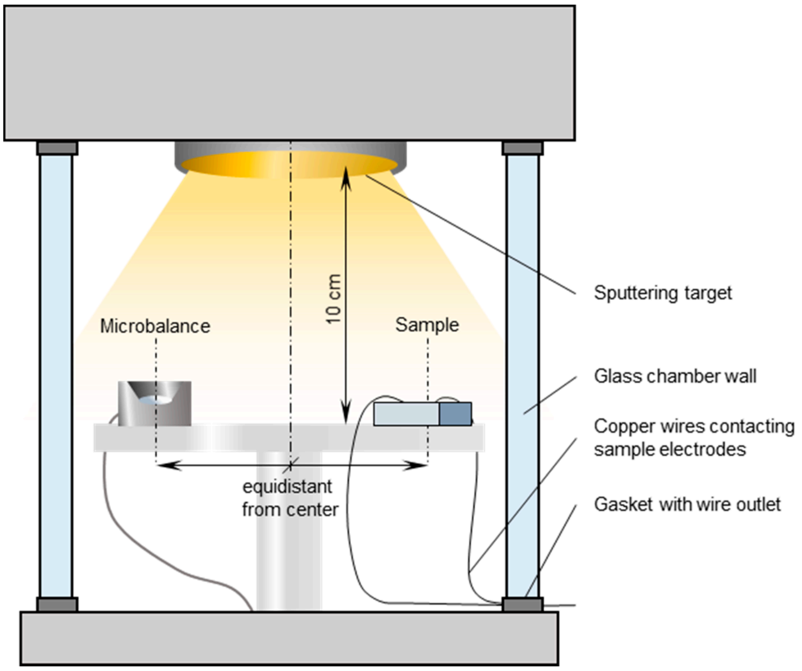

Figure S1. Setup of the sputter deposition chamber for in-situ resistance measurements.

2. Contact Angles and SFE Derivation

The surface free energies of the different metals and the polymer surface was estimated by static contact angle measurements. From the contact angles of the three test liquids with the surface obtained for each material as listed in **Error! Reference source not found.** the surface free energy was obtained as three components by solving for the resulting system of three equations for the unknown quantities. For the respective wetting liquid, the contact angle is related to the energy components of the tested surface as follows:

$$\gamma_L(\cos\theta + 1) = 2\left(\sqrt{\gamma_L^{LW}\gamma_S^{LW}} + \sqrt{\gamma_L^-\gamma_S^+} + \sqrt{\gamma_L^+\gamma_S^-}\right)$$

where θ is the contact angle, σ denotes the respective surface energy component for the liquid (index L) or the surface (index S), LW indicates the dispersive, Lifshitz–van der Waals component while + stands for the positive polar and − for the negative polar component. The quantity γ_L is related to the liquid surface energy components by

$$\gamma_L = \gamma_L^{LW} + 2\sqrt{\gamma_L^+\gamma_L^-}.$$

Using three different test liquids with known surface energy three equations can be obtained:

$$\gamma_{L1}(\cos\theta_{L1} + 1) = 2\left(\sqrt{\gamma_{L1}^{LW}\gamma_S^{LW}} + \sqrt{\gamma_{L1}^-\gamma_S^+} + \sqrt{\gamma_{L1}^+\gamma_S^-}\right)$$

$$\gamma_{L2}(\cos\theta_{L2} + 1) = 2\left(\sqrt{\gamma_{L2}^{LW}\gamma_S^{LW}} + \sqrt{\gamma_{L2}^-\gamma_S^+} + \sqrt{\gamma_{L2}^+\gamma_S^-}\right)$$

$$\gamma_{L3}(\cos\theta_{L3} + 1) = 2\left(\sqrt{\gamma_{L3}^{LW}\gamma_S^{LW}} + \sqrt{\gamma_{L3}^-\gamma_S^+} + \sqrt{\gamma_{L3}^+\gamma_S^-}\right).$$

The three unknown quantities of the tested surface γ_S^{LW}, γ_S^+ and γ_S^- can therefore be found by using simple linear algebra.

The used surface energy components of the test liquids [1] are listed in **Error! Reference source not found.**.

Table 1. Contact angles of the three test liquids with the respective surface given in degrees. Values are averages of at least ten independent measurements and standard deviations are indicated.

Test liquid	Silver			Gold			Copper			Nickel			Polymer		
Formamide	46.4	±	1.6	57.2	±	1.2	42.0	±	2.5	24.6	±	1.0	69.8	±	2.2
Glycerol	65.1	±	1.1	62.8	±	1.3	47.5	±	1.4	41.8	±	0.5	78.2	±	1.0
Water	71.4	±	3.7	57.3	±	1.2	28.3	±	0.7	30.4	±	1.5	84.9	±	1.6

Table 2. Surface energy components of test liquids according to Ref. [1].

Test liquid	γ_L	γ_L^{LW}	γ_L^-	γ_L^+
	mJ m^{-2}			
Formamide	58.2	35.6	65.7	1.95
Glycerol	63.9	34.4	12.9	16.9
Water	72.8	21.8	10	65

3. Kratky analysis

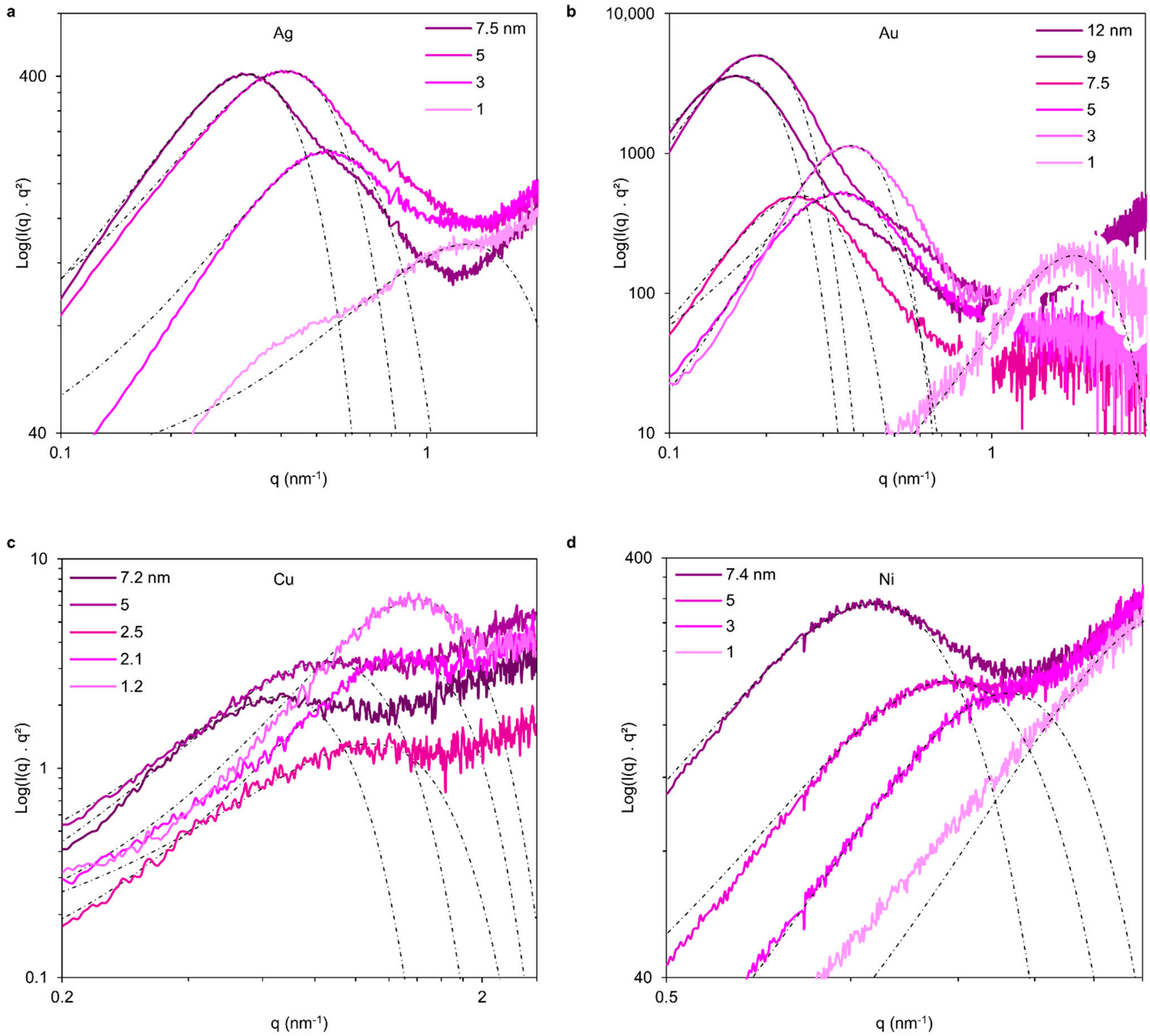

Figure S2. Kratky plots of the scattering curves obtained from cuts along the Yoneda horizon of the scattering patterns with fitted gaussian functions indicated by dashed lines. (**a**) Silver (**b**) Gold (**c**) Copper (**d**) Nickel.

4. Experimental Pair Distance Distribution Computation

For the calculation of the pair distance distributions we evaluated line cuts along the y-axis (Figure 4a,b) at q_z positions of the Yoneda horizon where the intensity has its maximum. We follow the argument of Lazzari [2] and neglect changes in the gradient of the dielectric index. We avoid the limitations of spherical or cylindrical form factors and reformulate the working equation given by

$$I(Q) \propto |\mathcal{F}_2(\rho(r_y))[Q]|^2 \times \mathcal{F}_2(p(r_y))[Q]$$

to

$$I(Q) \propto \mathcal{F}_2(PDD(r_y))[Q]$$

because we are working with the in-plane scattering (parallel to the surface) along q_y. The Furier transform is also given by

$$\mathcal{F}_D(p(r))[Q] = \int_0^\infty drp(r)J_{D/2-1}(Qr)/(Qr)^{D/2-1}$$

while for two dimensions D = 2 and is obtainable from the Bessel zero transform of the pair distance distribution of scattering sites in plane. It is then explicitly given by

$$\mathcal{F}_2(PDD(r_y))[Q] = \int_0^\infty dr_y\, PDD(r_y)\, J_0(Qr_y).$$

Using the Bessel zero transform to obtain the Fourier transform we minimized the L^2 norm according to

$$\min \parallel I(Q) - \mathcal{F}_2(PDD(r_y))[Q] \parallel_2$$

thereby computing an experimental pair density distribution. The obtained optimal fits can be regarded as the real space equivalents of the scattering intensity data. For interpretation, the pair distance distributions calculated for arbitrary geometrical objects can be matched to the experimental pair distance distributions (see Section 0).

5. Experimental Pair Distance Distributions

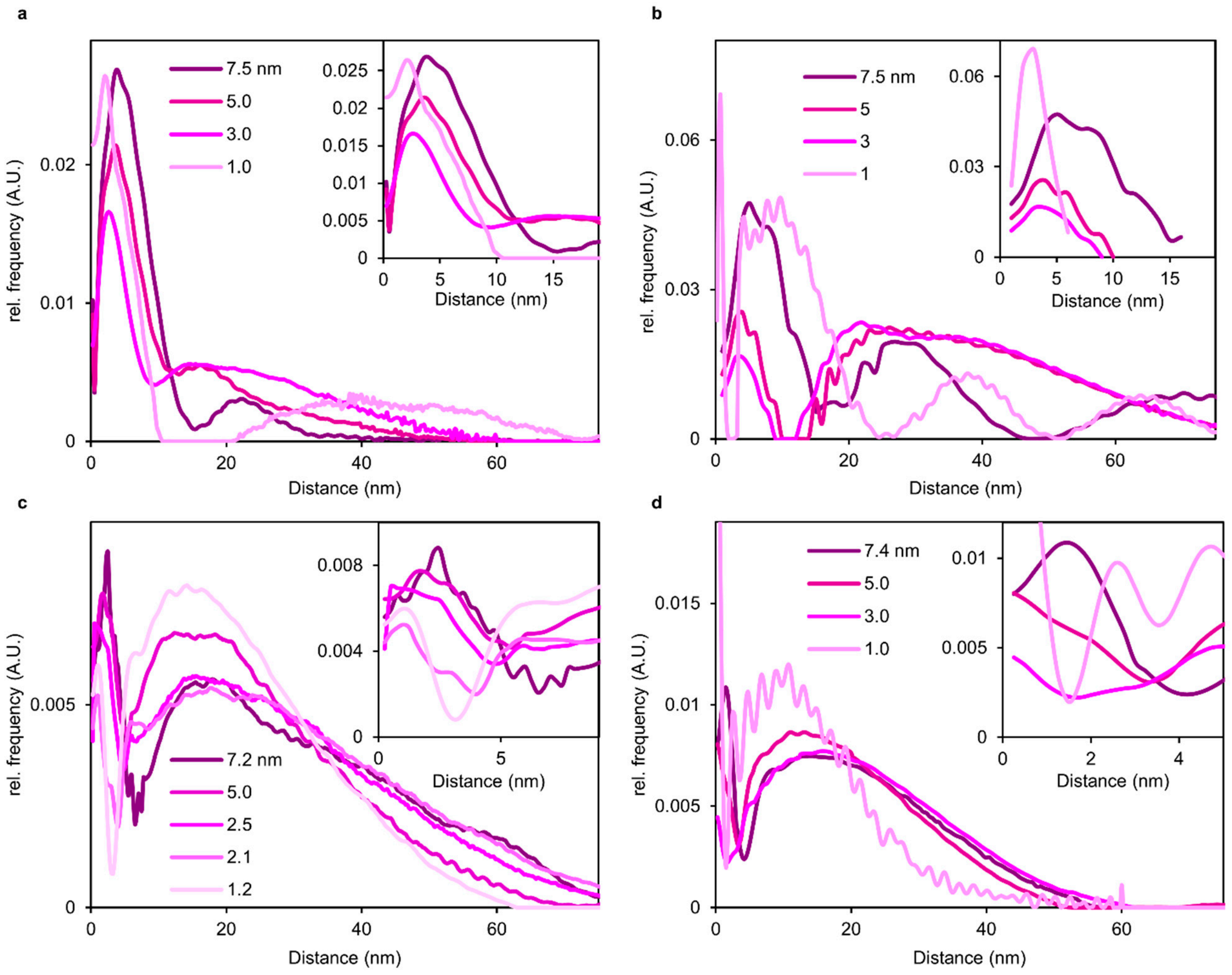

Figure S3. Pair distance distributions as fitted to the raw qy-data of the different metal thin films. The insets are plots of the first peaks of the respective pair distance distributions for better visibiliity of the shifting maxima. (**a**) Silver (**b**) Gold (**c**) Copper (**d**) Nickel.

6. qy-cuts and Scattering Curves Reconstructed from PDDs

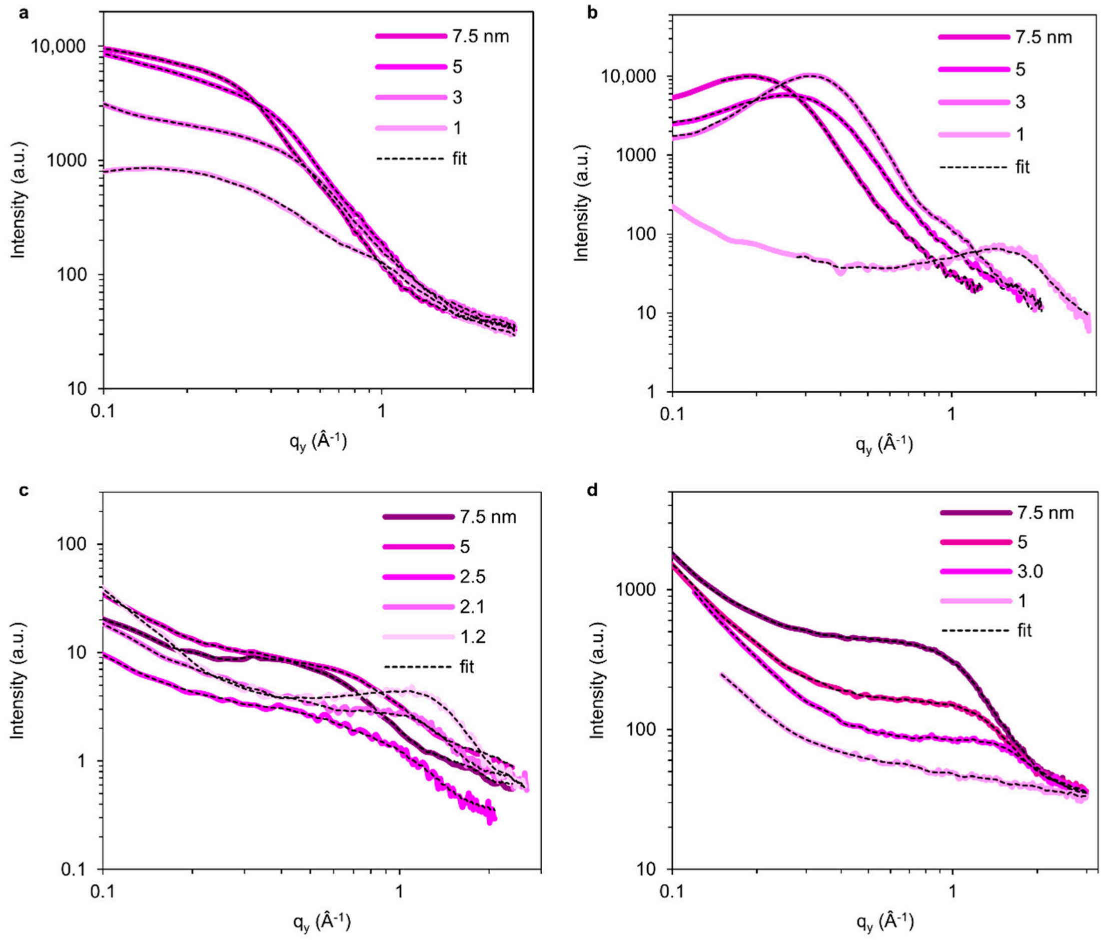

Figure S4. Raw data of the q_y-cuts along the Yoneda horizon for the different metal films (colored lines) and the scattering patterns as reconstructed from the respective fitted pair distance distributions (dashed lines). (**a**) Silver (**b**) Gold (**c**) Copper (**d**) Nickel.

7. Pair distance distribution calculation and fitting

Pair distance distribution functions (PDDs) were calculated, fitted and visualized with the Mathematica 11.3 software package.

For the numerical calculation of PDDs the geometric region for which the distribution should be calculated is defined (e.g., a spherical cap or an ensemble of spheres on a lattice) as a first step (**Error! Reference source not found.**a). This region is then randomly filled with 5×10^3 to 10^4 points using the innate function "RandomPoint" as visualized in **Error! Reference source not found.**b and c for the region of interest simulating potential scattering sites. For each individual point the distances to all other points in the x-y plane were calculated and binned to the desired resolution ultimately yielding the pair distance distribution of the defined region in the q_y-direction.

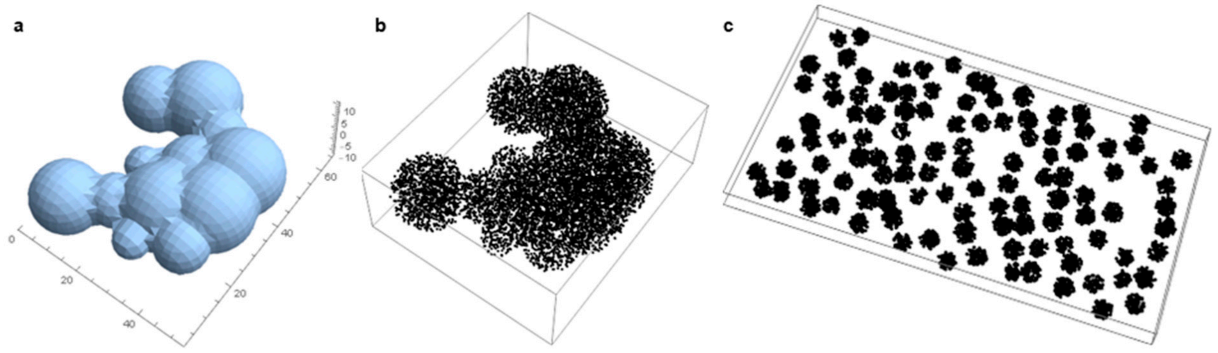

Figure S5. Region defining (**a**) and generating random points for numerical PDD calculation of cluster shapes (**b**) and arrangements (**c**).

To find cluster shapes, sizes and arrangements that fit the experimental PDDs (as obtained from the fits to the raw scattering data) two approaches were chosen. Either, the shape and size of the cluster was selected and adjusted manually which was primarily done with spherical cap geometries to estimate parameters for the second method and to illustrate the inadequacy of these geometries. On the other hand, a simple approach of using the best fitting structures from a set of randomly generated assemblies as an estimate for the average of cluster shape was implemented. A set of 2000 random model clusters were generated for each cluster shape analysis. The quality parameter whereby the assemblies were rated was chosen as the root mean square deviation of the simulated PDD and the experimental PDD in the region of interest. Cluster shape and size was modelled with a 4 by 4 hexagonal lattice occupied to a random extent (between 25 and 60%) with spheres of either equal or randomly (normal) distributed size (as visualized in **Error! Reference source not found.**a. The resultant model clusters were characterized in terms of size using the radius of gyration (R_g). For non-spherical footprint clusters (the majority), for comparability with spherical clusters an equivalent radius (R_{eq}) was calculated as the radius of a sphere with identical R_g. Circles with these radii are indicated in the top-down projections and R_{eq} is reported alongside. Finally, the cluster shape was characterized with the parameter ε, the ratio of cluster footprint within the R_{eq}-circle to the area of that circle. This parameter indicates how elongated and branched the cluster structure is.

Arrangements of clusters on the surface were fit on lattices of different size (depending on the purpose) with lattice points being randomly displaced to a certain extent. The occupancy of the lattice with spheres was randomly generated within a specified range whereas the spheres were either monodisperse or their size was subject to a uniform random distribution within a specified range.

8. Pair Distance Distribution Fits and Morphology

8.1. Silver

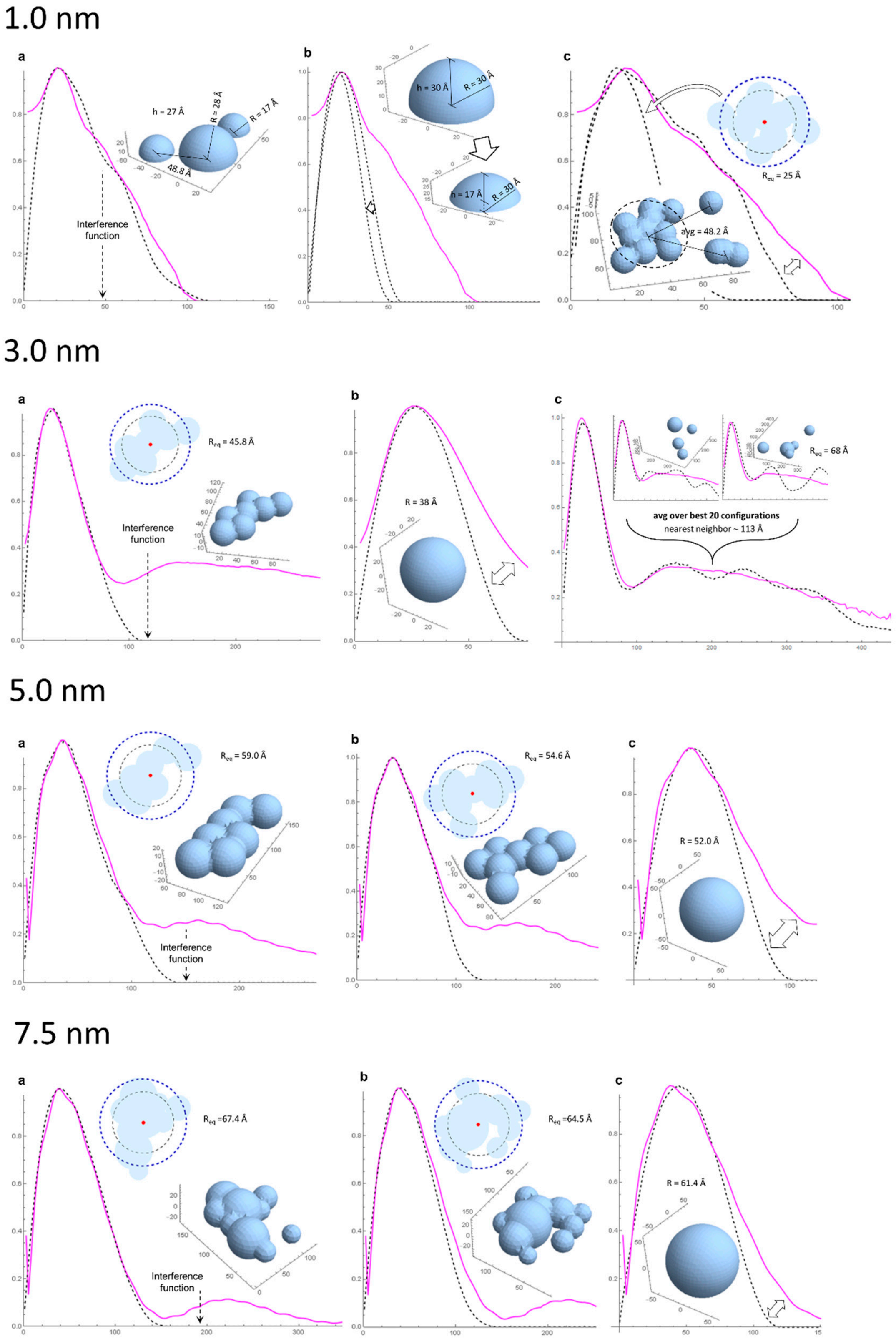

Figure S6. Pair distance distribution function fits for silver thin films with equivalent film thicknesses of 1.0, 3.0, 5.0 and 7.5 nm. Panels **c** for 1.0 and 3.0 nm show fits taking nearest neighbors into account. The remaining panels show fits to the first peaks to obtain cluster shapes. y-axes are in arbitrary units and x-axes are in Â.

The PDDs obtained for thin films of silver show pronounced peaks located at distances ranging from ~20 Â to 45 Â. Except for the data obtained for 1.0 nm thickness, the first peak is followed by a second peak that coincides with the average cluster-to-cluster distance extracted with Kratky analysis (stemming from the interference function) as indicated by the dashed arrow in **Error! Reference source not found.** panels a. In the data for 1.0 nm thickness a shoulder is observable at the interference function distance and no further maximum is present except for a very broad peak located at very long distance of ~400 Â starting at ~200 Â. Thus, it seems that a short-range order is not strongly established which is also hinted at by the small peak in the Kratky plot from the data of 1.0 thickness. Neglecting the contributions of nearest neighbors, the cluster shape can be approximated by a spherical cap geometry with a radius and a height of 30 Â (**Error! Reference source not found.** 1.0 nm b). However, if the unit cell approximation (Figure 4c) and the corresponding mass balance is invoked, the spherical cap must have a height of only 17 Â which shifts the PDD towards lower distances worsening the fit (as indicated by arrows in **Error! Reference source not found.** 1.0 nm b). Therefore, the cluster shape is better approximated by a somewhat elongated structure (**Error! Reference source not found.** 1.0 nm c). By introducing several small neighbors, the contributions at larger distances can be accounted for. A more complete model with neighbors at further distances could eliminate the discrepancy indicated by the double arrow in **Error! Reference source not found.** 1.0 nm c. As indicated above, the remaining PDDs have the interference function peak separated from the cluster structure peak. The equivalent radii of the estimated clusters increase with increasing film thickness from ~45 to ~65 Â but cannot be approximated by a spherical shape due to considerable discrepancies at distances larger than the peak maximum (arrows in the panels b, c and c of 3.0, 5.0 and 7.5 nm, respectively). The cluster morphology transitions from quite spherical (1.0 nm) over elongated (3.0 nm) to somewhat spread out (5.0 nm) and frayed (7.5 nm). This is in accordance with the notion of small spherical clusters growing to touch and coalesce to a point where incomplete coalescence leads to the percolation into a network of irregular clusters that gets filled gradually. This filling is illustrated by the fact that the structures obtained for 7.5 nm have a more compact and spherical footprint than the elongated ones obtained for 3.0 and 5.0 nm thickness.

In general, the PDD fits obtained for silver display quite low relative frequencies at distances beyond the minimum separating the cluster from the order peak. This can be ascribed to a weak correlation with neighboring clusters due to a relatively random arrangement on the polymer surface. This is also in line with the q_y data of silver displaying the least pronounced interference function features. Therefore, no model arrangement could be fitted to the data which covers more than a few neighbors without having substantial discrepancies. However, fitting an arrangement of several neighbors yielded distances which coincided very well with the inter-cluster distances derived from Kratky-analysis (**Error! Reference source not found.** 1.0 nm c and 2.0 nm c).

8.2. Gold

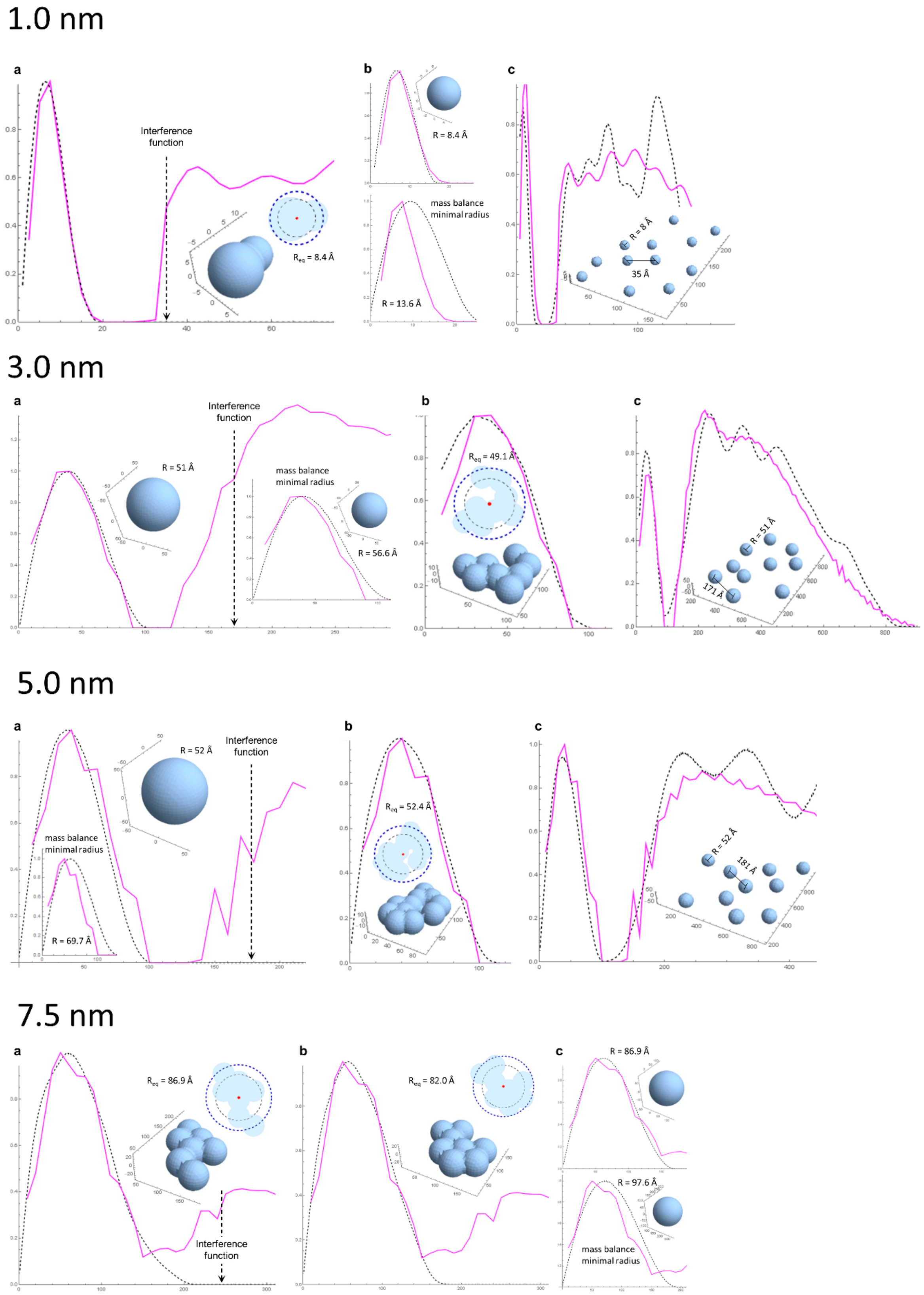

Figure S7. Pair distance distribution function fits for gold thin films with equivalent film thicknesses of 1.0, 3.0, 5.0 and 7.5 nm. Panels **a** and **b** show fits to the first peaks to estimate cluster shape for all thicknesses. Panel **c** shows arrangement fits for thicknesses 1.0, 3.0 and 5.0 while panel **c** of 7.5 nm shows PDDs of spherical clusters for reference. y-axes are in arbitrary units and x-axes are in Å.

The PDDs obtained for gold are characterized by peaks located at relatively low distances ranging from 7.5 Â to approximately 60 Â and are well separated by a substantial minimum from the spread-out peaks of the nearest neighbors (**Error! Reference source not found.** panels a). For all PDDs obtained, the location of the nearest neighbor order peak agrees well with the interference function distance. The first peaks of all distributions can be fitted reasonably well with a spherical cluster although there are some subtle discrepancies. The 1.0 nm data can be best approximated (**Error! Reference source not found.** 1.0 nm b) but a slightly elongated cluster fits better (**Error! Reference source not found.** 1.0 nm a). The peaks in the data for 3.0 nm and 5.0 nm thickness displays a sharp drop to 0 at 90 Â and 100 Â, respectively, that cannot be accounted for by the PDD of a spherical cluster. The clusters resulting from the fit procedure are somewhat elongated and exhibit a sharper decline at long distances than a spherical shape (**Error! Reference source not found.** 3.0 and 5.0 nm b) although the fit for 3.0 nm is worse at small distances. Similarly, the first peak of the PDD corresponding to 7.5 nm film thickness is best estimated by elongated clusters (**Error! Reference source not found.** 7.5 nm a and b). However, notice that all estimated irregular cluster are still the closest to a spherical footprint of all investigated metals. Since the cluster shape for all thicknesses is very close to spherical it was interesting to apply the unit cell approximation with the associated mass balance. PDDs obtained for spherical clusters with the minimal radius implied by mass balance are indicated by "mass balance minimal radius" in **Error! Reference source not found.**. Interestingly, the cluster sizes suggested by this approach display PDDs with substantial discrepancies to the actual peaks as they are generally overestimated. This suggests that the mass balance is either closed by small clusters interspersed randomly between the larger islands or that the cluster distance is overestimated or a combination of both.

Larger arrangements of clusters were fitted to the PDDs from 1.0 nm to 5.0 nm. The PDD for 7.5 nm does not allow a meaningful fit of an arrangement since contributions of distances in the range of the nearest neighbor are quite low. This is likely the result of both, the resolution limit of the setup and an increasingly disordered arrangement of the scattering centers. The arrangement fit to the data of 1.0 nm thickness displays order peaks that coincide very well with the data corroborating the distance of 35 Â found with Kratky-analysis. The order peaks for 3.0 and 5.0 thickness vanish gradually indicating a more random arrangement due to incomplete coalescence with the consequential elongation and branching. However, the general shape is very well represented by the arrangements where the cluster distance was set to the values deduced from Kratky-analysis (171 Â and 181 Â).

8.3. Copper

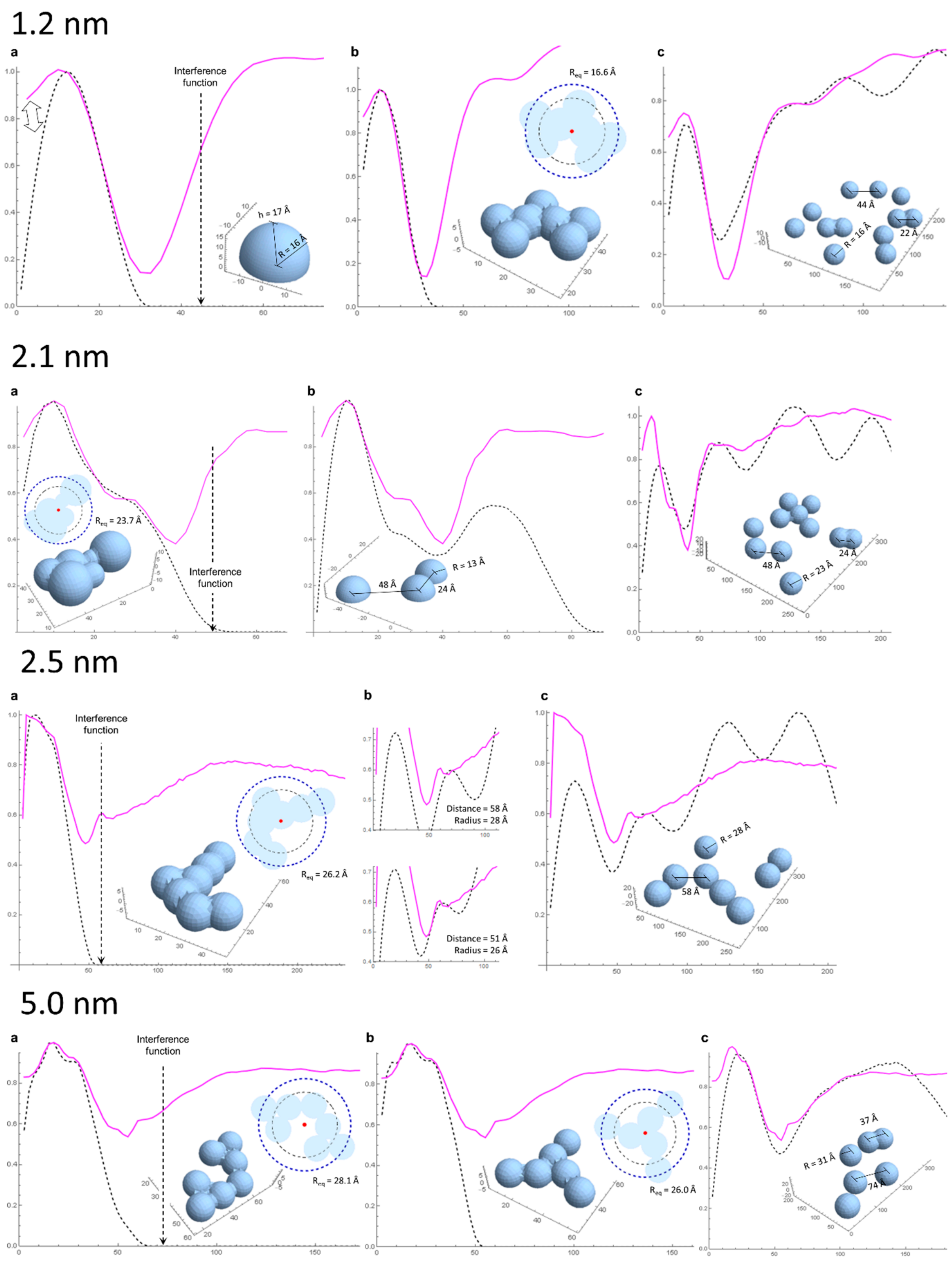

Figure S8. Pair distance distribution function fits for nickel thin films with equivalent film thicknesses of 1.2, 2.1, 2.5 and 5.0 nm. Panels **a** and panels **b** of 1.2 and 5.0 nm show cluster shape fits. Panel b of 2.1 nm shows a spherical cap geometry approximation with two nearest neighbors. Panels **c** show arrangement fits for the respective nominal thicknesses. Panel **b** for 2.5 nm illustrates the difference in PDD resulting from adjusting the average cluster distance from 28 to 26 Å. y-axes are in arbitrary units and x-axes are in Å.

All PDDs fit to the q_y-data of copper exhibit a pronounced peak located at relatively low distances (roughly 15–20 Â) followed by a second peak that coincides, at least to some extent, with the inter-cluster distance estimated from Kratky analysis (**Error! Reference source not found.** panels a). These two maxima are separated by a minimum (roughly 30–60 Â). Although the first peaks can be approximated to a certain extent by spherical clusters considerable deviations are present even for the smallest clusters (**Error! Reference source not found.** 1.2 nm a indicated by the double arrow). The discrepancy is especially pronounced at the shortest distance contributions. This shows that even at an equivalent thickness of 1.2 nm the clusters are not relaxing into spherical equilibrium shapes but are non-spherical to some extent. Accordingly, the cluster morphologies which were obtained from the fits to the first peaks are markedly non-spherical with a trend of more elongated and branched morphology with increasing film thickness. Additionally, and as expected, the cluster sizes increase with increasing deposition thickness from an equivalent sphere radius of 16.6 Â to ~27 Â. The most elongated shape was estimated for the clusters at a thickness of 2.5 nm which coincides with the onset of percolation. The cluster morphology for 5.0 nm is more compact which is likely due to the formation of more contact points with neighbors and the filling of holes after percolation.

The arrangement of the clusters on the surface was fit to a larger portion of the PDDs with a larger lattice. From a smallest allowed inter-cluster distance half of that inferred from Kratky analysis the fits converged to arrangements (**Error! Reference source not found.** panels c) with cluster distances coinciding with the respective interference function distance (with small deviations such as in **Error! Reference source not found.** 2.5 b). Judging from the size of the order peaks in the PDDs compared to the fits and the absence of pronounced next neighbor peaks the arrangements of clusters on the polymer surface seem to be quite disordered. Additionally, for higher equivalent thicknesses the order peaks are less pronounced presumably due to the branching and percolation of the clusters. Also, the presence of small clusters in between the larger percolating islands could contribute to a more broad distribution of distances.

8.4. Nickel

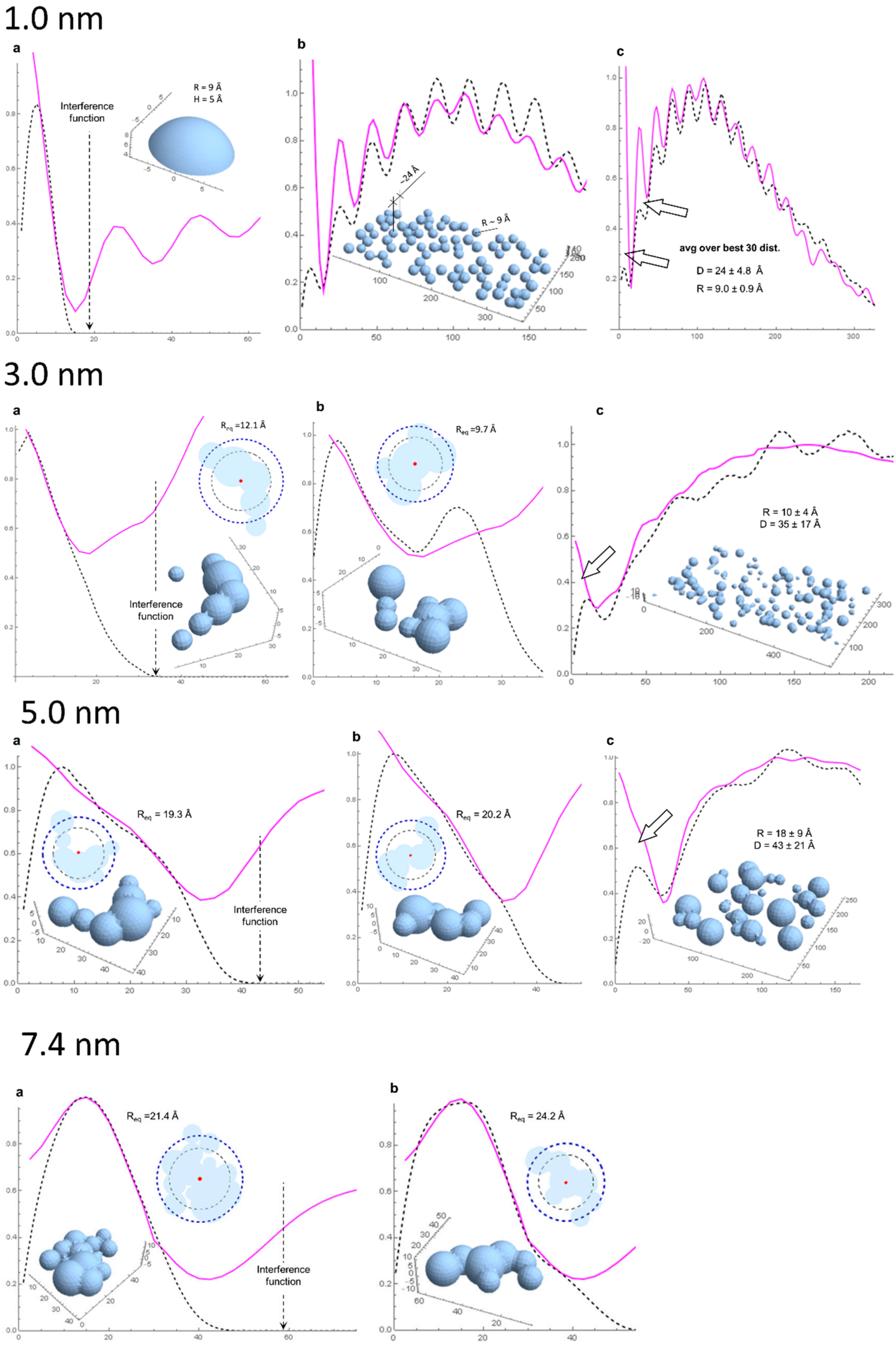

Figure S9. Pair distance distribution function fits for nickel thin films with equivalent film thicknesses of 1.0, 3.0, 5.0 and 7.4 nm. Panels **a** as well as **b** for 3.0, 5.0, and 7.4 nm show cluster shape fits while panel **b** of 1.0 nm and panels **c** for 3.0 and 5.0 nm show arrangement fits for the respective nominal thickness. Panel c for 1.0 nm shows the average of the respective 30 best arrangement fits. y-axes are in arbitrary units and x-axes are in Å.

The PDDs obtained from the fits to the q_y-data of nickel do not exhibit peak shaped maxima for the small distance contributions apart from the one obtained for an equivalent film thickness of 7.4 nm (**Error! Reference source not found.** panels a). They rather show a maximum at the smallest distance and a following steady decrease towards a minimum located at distances between 15 and 43 Â. For the lowest thickness of 1.0 nm the PDD of a spherical cap shape with a radius of 9 Â and a height of 5 Â can mostly account for the slope of the PDD indicating compact metal island shape. However, the mass balance requires substantially larger clusters (13 Â) which could be satisfied by even smaller clusters nucleating in the regions between the larger ones. Thus, also the discrepancy at the smallest distances could be accounted for. For the thicknesses 3.0 and 5.0 nm the slope cannot be approximated with spherical clusters and elongated or somewhat branched cluster morphologies resulted from the fits. Also, very small clusters which are not in contact with the large one but in close proximity can contribute to the small distances **Error! Reference source not found.** 3.0 nm a and b. This thickness of 3.0 nm is very close to the onset of percolation and therefore the shape estimations resulting from the fits are quite intuitive as neighboring clusters barely touch through contacts with small, interspersed islands. Equivalent radii of the fitted structures increase from 9 to 24 Â and the shape of the clusters also increases in irregularity from a quite spherical approximation to a more frayed or branched appearance. For a thickness of 7.4 nm the inferred cluster structure can be thought of as resulting from the hole filling process happening after percolation. Thus, the structures are still somewhat branched but less elongated and more compact.

The overall shape of the PDDs was fitted only for thicknesses up to 5.0 nm. Best fits were obtained for arrangements where the average inter-cluster distance was very similar to that obtained from Kratky analysis. In general, the arrangements could be fitted reasonably well with minor discrepancies (arrows in **Error! Reference source not found.** panels c). The discrepancy at the first peak is most probably the result of approximating the clusters with a spherical rather than an elongated or branched shape so that small distance contributions from 'offshoots' are not represented. Furthermore, the second peak representing the nearest neighbor order in **Error! Reference source not found.** 1.0 nm c cannot be accounted for sufficiently by the fitted model. This could be caused by neglecting small clusters interspersed between the large ones. The strong oscillations of the PDD data could also be an artifact to some extent resulting from fact that only a finite window of q_y-data is available for PDD fitting. Nevertheless, the PDD shapes and the corresponding fit parameters demonstrate that the arrangement as well as the size and shape distributions get increasingly disordered and polydisperse. Especially the difference between the data of 1.0 and 3.0 nm is striking and most likely due to transition from the nucleation to the coalescence regime (see normalized scattering data analysis).

9. Supplementary Table

Table S3. Contact angles of test liquids with metals after surface exposure to ambient air for 24 hours.

Test liquid	Silver			Gold			Copper			Nickel		
Formamide	26.0	±	1.6	56.7	±	1.7	27.8	±	4.5	20.6	±	2.1
Glycerol	75.9	±	0.4	72.8	±	2.9	58.2	±	1.4	36.6	±	4.8
Water	79.3	±	1.1	82.1	±	0.4	57.9	±	0.7	34.5	±	1.6

References

1. Volpe, C. D.; Siboni, S. Acid–Base Surface Free Energies of Solids and the Definition of Scales in the Good–van Oss–Chaudhury Theory. *J. Adhes. Sci. Technol.* **2000**, *14*, 235–272, https://doi.org/10.1163/156856100742546.
2. Lazzari, R.; Renaud, G.; Revenant, C.; Jupille, J.; Borensztein, Y. Adhesion of Growing Nanoparticles at a Glance: Surface Differential Reflectivity Spectroscopy and Grazing Incidence Small Angle x-Ray Scattering. *Phys. Rev. B* **2009**, *79*, 125428, https://doi.org/10.1103/PhysRevB.79.125428.
